# Two heterologously expressed *Planobispora rosea *proteins cooperatively induce *Streptomyces lividans *thiostrepton uptake and storage from the extracellular medium

**DOI:** 10.1186/1475-2859-9-44

**Published:** 2010-06-09

**Authors:** Anna Giardina, Rosa Alduina, Elvira Gottardi, Valentina Di Caro, Roderich D Süssmuth, Anna M Puglia

**Affiliations:** 1 Dipartimento di Biologia Cellulare e dello Sviluppo, Università di Palermo, Viale delle Scienze, Ed.16, 90128 Palermo, Italy; 2 Technische Universität Berlin, Strasse des 17. Juni 124, 10623 Berlin, Germany; 3 Fondazione RiMed, Piazza Sett'Angeli 10, 90134 Palermo, Italy

## Abstract

**Background:**

A bacterial artificial chromosomal library of *Planobispora rosea*, a genetically intractable actinomycete strain, was constructed using *Escherichia coli*-*Streptomyces *artificial chromosome (ESAC) and screened for the presence of genes known to be involved in the biosynthesis of antibiotics.

**Results:**

One clone with a 40 kb insert showed antimicrobial activity against Gram positive bacteria. Insert sequence analysis and subcloning experiments revealed that the bioactivity was due to a 3.5 kb DNA fragment containing two open reading frames. These *orfs *encode two proteins with high similarity to a putative membrane protein of *Streptomyces coelicolor *and to the nogalamycin resistance protein SnorO of *Streptomyces nogalater*, respectively. The role of these two Orfs is unknown in *Planobispora. *Disruption and complementation experiments revealed that both proteins are necessary for the antibacterial activity and chemical analysis demonstrated that the antibiotic activity was due to thiostrepton, antibiotic used as recombinant clone selection marker.

**Conclusion:**

Two *Planobispora rosea orfs *are responsible for increasing intracellular amounts and storage of thiostrepton in *Streptomyces lividans*.

## Background

Thiostrepton is a potent thiopeptide antibiotic, widely used as a selection marker for thiostrepton-resistant (*tsr*)-vectors in *Streptomyces lividans *but its routine use revealed several unexpected biological activities. Remarkably, it can induce resistance to antibiotics having different cellular targets including daunorubicin, sparsomycin, tetranactin and GE2270A [1] and causes expression of thiostrepton-induced proteins [2]. Two of these thiostrepton-induced proteins, TipAL and TipAS, were demonstrated to be alternative in-frame translation products of the *tipA *gene [3]. In particular, they share the C-terminal region containing the binding domain for thiostrepton and similar cyclic thiopeptide antibiotics [1]. Notwithstanding TipA was initially assigned as a thiostrepton-induced activator of its own transcription [1], its role has not been deeply understood yet. In fact, *tipA *has been found to be present in many non-thiopeptide producing strains [4] and it is unclear what metabolic signals are the genuine inducers of its expression. Recently, it was established that thiostrepton is derived from the precursor TsrA, a genetically encoded peptide, suggesting that thiopeptide antibiotics are ribosomally synthesized [5-8].

Heterologous expression systems were successfully applied to synthesize metabolites, produced by hard-to-manipulate strains [9]. Previous results [10-12] have shown that *E. coli*-*Streptomyces *artificial chromosomes (ESACs), carrying large inserts of actinomycete DNA, can be introduced into a genetically accessible strain such as *Streptomyces lividans*, where they are stably maintained as an integrated form in its chromosome.

*Planobispora rosea *is a genetically intractable actinomycete producer of the thiazolylpeptide antibiotic GE2270, a potent inhibitor of the bacterial elongation factor Tu, which is structurally similar to thiostrepton [13].

In this paper we show that two *P. rosea orfs*, encoding a membrane protein and an ABC transporter, when cloned in *Streptomyces lividans*, determine thiostrepton uptake and storage from thiostrepton-containing medium. This has been experimentally shown by HPLC-ESI-mass spectrometry, HPLC-UV-DAD, feeding experiments with ^13^C-labeled cysteine and bioassays.

## Results

### Cloning and sequence analysis of a *Planobispora rosea *40 kb DNA fragment

**Figure 1:**
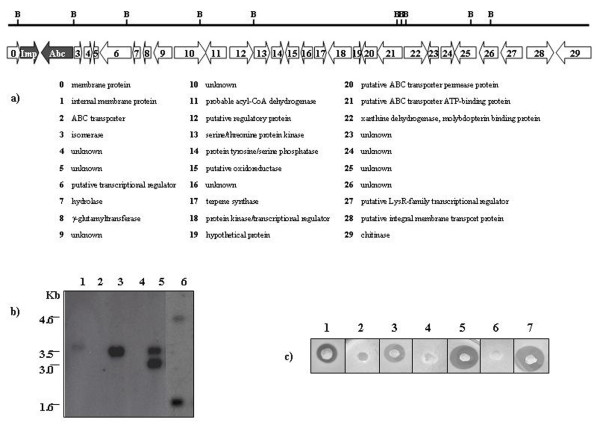
**Sequence analysis of the SL-40 insert and Bioassay**. a) Schematic diagram of sequence analysis of clone SL-40. Putative ORFs are indicated by the arrows; below is a list of the protein which each ORF is most similar to. The genes involved in SL-3.5 antibacterial activity are shown in grey and the remaining ORFs are in white. B indicates *Bam*HI sites. b) Southern blot analysis of *Bam*HI-digested total DNA isolated from SL-3.5 (lane 1), *S. lividans *(lane 2), *P. rosea *(lane 3), SL-ESAC (lane 4), SL-Abc (lane 5) and SL-Imp (lane 6). The *Bam*HI fragment of 3.5 Kb (derived from digestion of ESAC3.5) was used as probe. c) Bioassay against *Micrococcus luteus*: 1) SL-40; 2) SL-ESAC; 3) SL-3.5; 4) SL-Abc; 5) SL-Abc/Imp; 6) SL-Imp and 7) SL-Imp/Abc.

Four clones of *Streptomyces lividans*, SL-40, -48, -85, -120, isolated from a *P. rosea *genomic library [11], showed antibacterial activity against *M. luteus*. Since SL-40, which carried the smallest insert (40 Kb), was still able to show antibacterial activity, this construct was used for the subsequent investigations. Therefore, the insert was sequenced (GenBank sequence accession number: EU908202). The sequence analysis revealed thirty putative ORFs (Fig. 1a): ten hypothetical proteins, four putative regulators, six proteins related to membrane transport (three ABC-transporters and three membrane proteins), a chitinase, an isomerase, a xanthine dehydrogenase, a terpene synthase, a Ser-Thr-kinase, a Tyr-Ser-phosphatase, a gamma-glutamyltransferase, an acyl-CoA dehydrogenase, a hydrolase and an oxidoreductase. Since sequence analysis did not reveal the presence of any gene assignable to an antibiotic biosynthesis pathway, different fragments of the 40 kb insert were subcloned into the ESAC vector and the obtained nine subclones were repeatedly analysed for antibacterial activity against *M. luteus*. Bioassay revealed that only one *Streptomyces lividans *subclone, named SL-3.5 and carrying a 3.5 kb fragment, showed antimicrobial activity. Furthermore, it was found that SL-3.5 mycelium inhibited also the growth of *Bacillus subtilis*, but not of Gram-negative bacteria, as *E. coli *or *Pseudomonas aeruginosa *(data not shown). Swiss-prot analysis of the insert sequence indicated that this DNA fragment contained two divergently transcribed *orfs*, named *abc *and *imp*. *imp *consists of 1023 bp and the deduced product is a polypeptide consisting of 340 amino acids with 50% similarity to a membrane protein of *S. coelicolor *(SCO5138). *abc *consists of 2430 bp and encodes a protein of 809 amino acids that shows high levels of similarity (80%) with an ABC-transporter of *Streptomyces nogalater *involved in nogalamycin resistance (SnorO).

### Both *imp *and *abc *are involved in antibacterial activity of SL-3.5

To investigate the role of Imp and Abc in stimulating antibacterial activity in *Streptomyces lividans*, disruptants containing solely *imp *or *abc *were generated by insertional inactivation and named SL-Imp and SL-Abc (Fig. 1b). Bioassays showed that the single knockout mutants were not active against *M. luteus*, indicating that the expression of both *imp *and *abc *is mandatory for antibacterial activity (Fig. 1c).

To confirm that the disruption of *imp *and *abc *genes was the sole reason for the loss of antibacterial activity, complementation mutants were generated and investigated using the antibacterial assay. Our results indicated that, comparably to SL-3.5, both complementation strains had a high inhibitory activity on *M. luteus *(Fig. 1c).

### The antibacterial activity of SL-3.5 is determined by thiostrepton

*Streptomyces lividans *ZX7 carries the actinorhodin, undecylprodigiosin and calcium-dependent antibiotic biosynthesis gene clusters in its genome, but they are produced only under particular nutritional conditions [14]. In the conditions we used (growth in R_2_YED for 4 days at 30°C) we did not detect any antibacterial activity of *S. lividans*. To investigate if *Planobispora *proteins were able to activate either *act *or *red *gene expression, spectrophotometric assays of SL-40, SL-3.5 and control strain (SL-ESAC) were carried out. Our results showed that the amount of Act and Red produced from SL-40 and SL-3.5 was equal to those produced by *S. lividans *and SL-ESAC (data not shown). Furthermore, SL-40 and SL-3.5 failed to produce the calcium dependent antibiotic CDA.

To identify the antibacterial substance, dried methanolic extracts of SL-3.5 were extracted with a mixture of water:acetone:ethyl acetate (1:1:1) and both phases analyzed by HPLC-ESI-MS analysis. The upper phase of SL-3.5 extract contained a large peak (t_R _= 7.0 min) which was absent in the SL-ESAC sample (data not shown). To our surprise, the UV-spectrum as well as the molecular mass ([M+H]^+ ^= 1664.5 Da) identified the active substance as thiostrepton (data not shown). Comparison with commercially available thiostrepton confirmed this result. Moreover, TLC analysis revealed that also the activity of SL-40 was exclusively due to thiostrepton (data not shown).

### SL-3.5 accumulates thiostrepton during antibiotic selection

As thiostrepton was used as selection marker for ESAC containing *S. lividans *strains and, at the time of this work, the thiostrepton gene cluster had not been identified yet, two hypotheses arose from our results. The first was the presence of silent thiostrepton biosynthetic genes in *S. lividans*, whose expression was activated by *Planobispora *proteins and the second one was the uptake and storage of thiostrepton from the medium during SL-3.5 growth under selection pressure and its subsequent release into thiostrepton-free medium.

**Figure 2:**
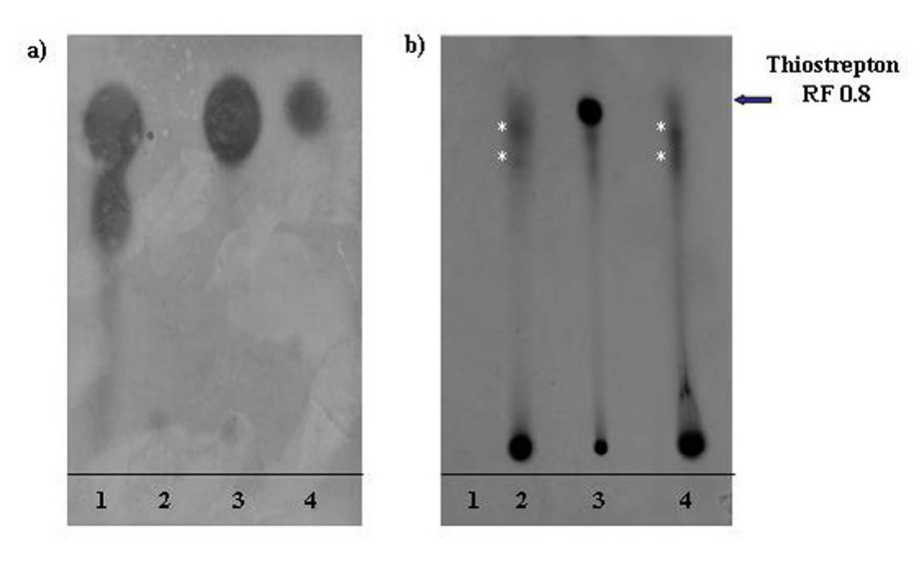
**Feeding experiments**. (a) Thin Layer Chromatography followed by bioassays on *M. luteus *and (b) autoradiography showing radio-labeled thiostrepton (1); SL-ESAC (2), *S. laurentii *(3) and SL-3.5 (4) radioactive extracts. Solvent: acetone. Asterisks indicate the substance present in SL-ESAC and SL-3.5 that are not active and are different from thiostrepton.

To investigate whether a *de novo *thiostrepton synthesis had occurred in *S. lividans*, we carried out feeding experiments using isotopically labelled (*R,S*)-[3-^14^C]cysteine as substrate. The radioactive samples were analyzed by TLC followed by bioassay and autoradiographic analysis. The bioassay clearly demonstrated that the inhibition halo generated by SL-3.5 on *M. luteus *corresponded to the TLC spot with R_f _= 0.8, identical to that one of thiostrepton produced from *S. laurentii *(Fig. 2a). However, the autoradiographic analysis revealed that only the antibiotic produced by *S. laurentii *is radioactive (Fig. 2b). Therefore, cysteine was not used by *S. lividans *as a substrate for the biosynthesis of the previously identified thiostrepton.

**Figure 3:**
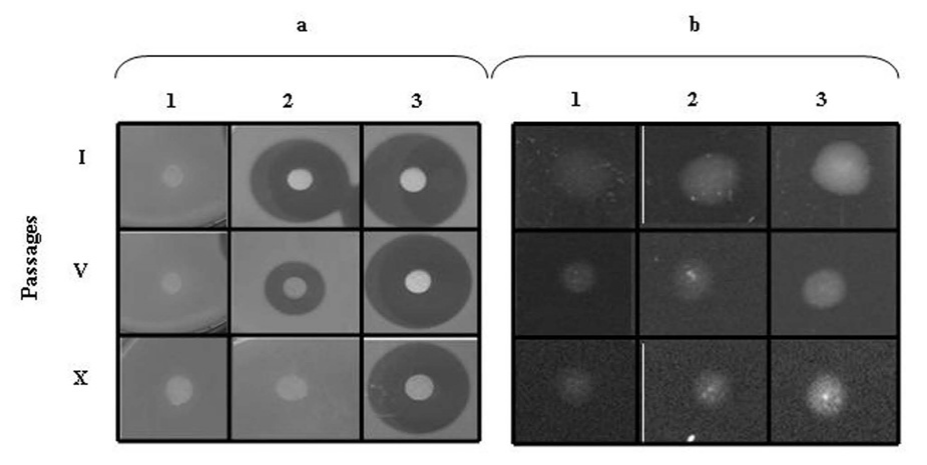
**Bioassay on *M. luteus *(a) and UV visualization (b): extracts were collected from SL-ESAC (1), SL-3.5 (2) and *S. laurentii *(3), grown for I, V or X passages in thiostrepton-free medium**.

In a subsequent set of experiments we investigated if the two *P. rosea *proteins, when present in SL-3.5, caused the uptake of the thiostrepton used for strain selection. Hence, we monitored the presence of the antibiotic in the mycelial DMSO extracts of SL-3.5 after one (I), five (V) and ten (X) passages of growth in thiostrepton-free R_2_YED medium by antibacterial bioassay (Fig. 3a). In addition, the same amount of each extract was spotted on a cover glass and photographed under UV light, as thiostrepton results luminescent (Fig.3b).

Thiostrepton was detected being released from SL-3.5 after passage I until passage V but could not be detected after passage X. As a positive control *S. laurentii *extracts were used and thiostrepton could always be detected upon extraction. As a negative control SL-ESAC was used, which was unable to take up and store thiostrepton.

All these results suggest that the thiostrepton is not synthesized *de novo *from SL-3.5 but it is accumulated in the cells from the thiostrepton-containing medium. The detected antibacterial activity is due to the release of thiostrepton during subsequent growth of SL-3.5 on thiostrepton-free medium.

### Thiostrepton enters the cells in SL-3.5

**Figure 4:**
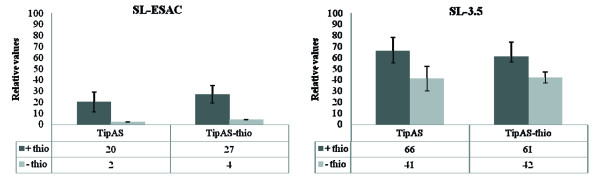
**Percentage volume of free and thiostrepton-bound TipAS forms**. SL-ESAC and SL-3.5 strains were grown with thiostrepton (+ thio) and for II passages without thiostrepton (- thio).

To investigate whether thiostrepton was membrane-bound or cytoplasmic, we analysed the expression level of TipAS and the occurrence of TipAS-thio (thiostrepton-bound TipAS) as Murakami *et al. *[3] demonstrated thiostrepton activates *tipA *expression and binds TipAS. Therefore, proteins extracted from SL-3.5 grown in presence or absence of thiostrepton for two passages in the culture medium were analyzed by 2D-DIGE and compared to the negative control SL-ESAC unable to store thiostrepton (Fig. 4). As expected [15], our results showed that expression of TipAS and TipAS-thio in the negative control SL-ESAC is 10- and 7-fold higher in presence of thiostrepton rather than in its absence. Interestingly, the protein analysis revealed that TipAS and TipAS-thio are 20- and 10-fold more expressed in SL-3.5 than in the negative control SL-ESAC grown in thiostrepton absence. In addition, externally added thiostrepton only slightly induced TipAS and TipAS-thio expression in SL-3.5, suggesting that thiostrepton was already present in the cells of SL-3.5 after the passage II in thiostrepton-free R_2_YED medium.

### Imp and Abc are involved in thiostrepton uptake

**Figure 5:**
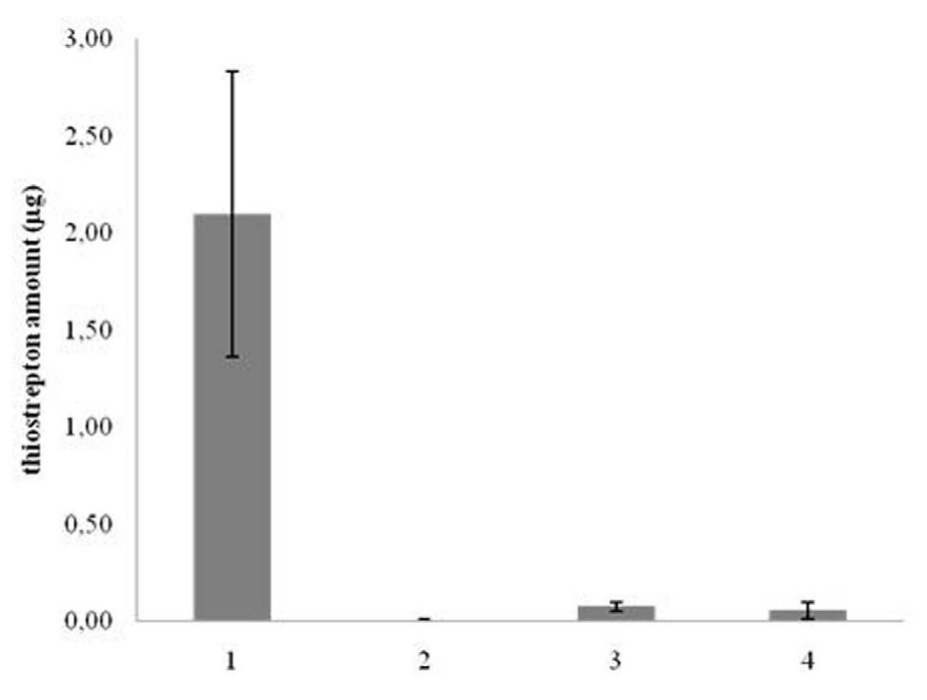
**HPLC analysis of thiostrepton: mycelium of SL-3.5 (1), SL-ESAC, (2), SL-Abc (3) and SL-Imp (4) grown for I passage without thiostrepton. The total amount of thiostrepton in the mycelium is given in μg/plate**.

In order to test if Imp and Abc are involved in thiostrepton uptake or secretion, we performed quantitative analysis by HPLC/UV-DAD/ESI-MS of mycelium extracts of SL-3.5, SL-Abc and SL-Imp after two passages in a thiostrepton-free medium. HPLC ESI-MS analysis revealed (Fig. 5) that both Orfs are essential for the uptake of the antibiotic, in fact while negative control SL-ESAC contained basically no antibiotic, SL-3.5 contained up to 2 μg of thiostrepton per plate and the two mutants SL-Abc and SL-Imp contained 0.07 and 0.05 μg per plate, respectively. Our results suggest that Imp and Abc are cooperatively involved in increasing the intracellular thiostrepton amount.

### Imp and Abc expression is medium-dependent and strain specific

**Figure 6:**
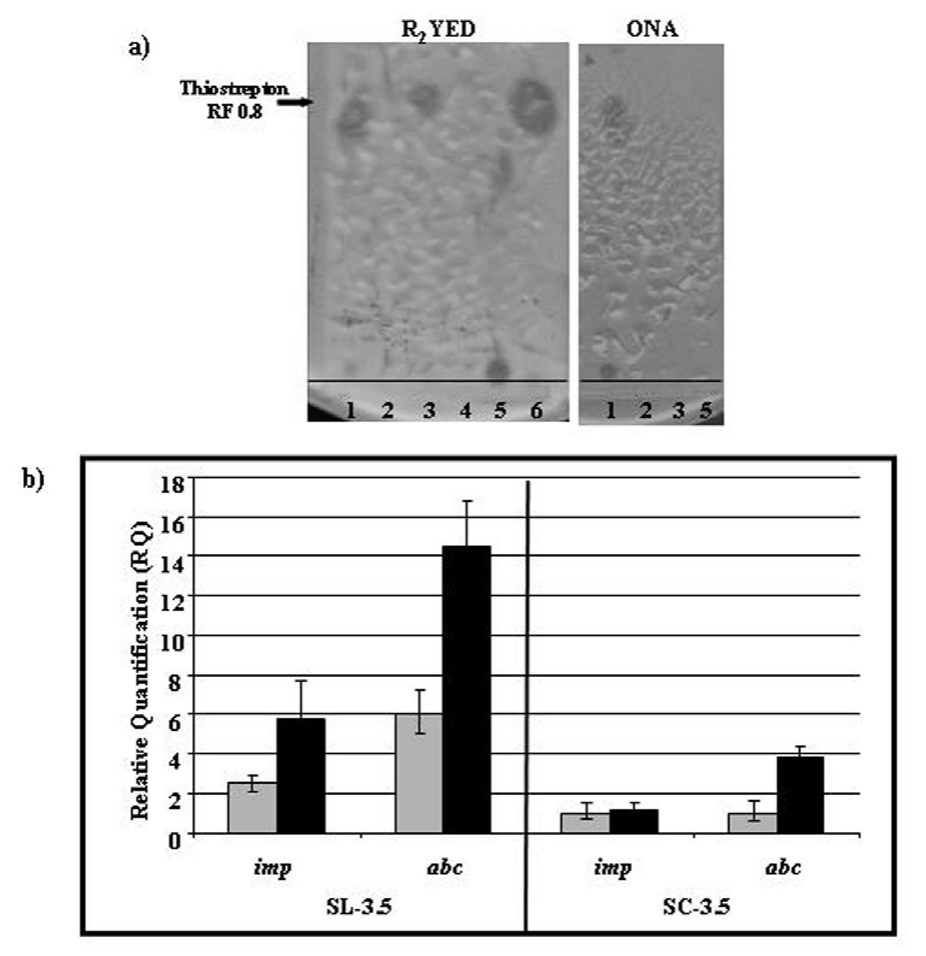
**The expression of *imp *and *abc *is medium-dependent and strain-specific**. a) Thin Layer Chromatography followed by bioassays of thiostrepton (1); SL-ESAC (2), SL-3.5 (3), *S. coelicolor *(4), SC-3.5 (5) and *S. laurentii *(6) extracts. Solvent: acetone. Tester strain: *M. luteus*. b) qRT-PCR analysis of *abc *and *imp *transcription in SL-3.5 and SC-3.5 grown on ONA (grey bars) and R_2_YED (black bars).

When SL-3.5 strain was cultured in various liquid or solid media different from R_2_YED (i.e. ONA), the antibiotic activity was never detected (Fig. 6a and data not shown) suggesting that the antibiotic effect could be medium-dependent. Thus, we investigated whether *imp *and *abc *genes were actively transcribed only in R_2_YED medium. Quantitative RT-PCR experiments were performed using total RNA extracted from SL-3.5 mycelium grown for 4 days on R_2_YED or ONA agar plates containing thiostrepton. The results clearly demonstrated that both genes are 2,4-fold more transcribed in SL-3.5 when grown in R2YED than in ONA (Fig. 6b). We can surmise that the expression levels of *imp *and *abc *were not enough to determine the uptake of thiostrepton in ONA.

In addition, we investigated whether other streptomycetes showed the same antibacterial effect when they contained these two genes. To this aim, we transformed *S. coelicolor *M145 with ESAC3.5, generating SC-3.5 strain. Its capacity to uptake and maintain thiostrepton was investigated by TLC analysis followed by bioassay of extracts. The findings showed that SC-3.5 extract does not contain thiostrepton (Fig. 6a). In accordance with this result, we found that the transcription level of *abc *and *imp *genes in SC-3.5 was lower than that of SL-3.5, even if *abc *transcription was 2-fold more induced in SC-3.5 grown in R_2_YED (Fig. 6b). All together these experiments suggest that thiostrepton uptake is medium-dependent and strain-specific.

## Discussion

Heterologous expression in genetically and physiologically characterized hosts has been used as tool to access the gene products of hard-to-manipulate strains [9]. *Streptomyces *species offer many potential advantages as hosts for the heterologous expression of secondary metabolite genes and we previously have successfully demonstrated heterologous expression of actinomycete genes in *S. lividans *[12].

At the beginning of this work, we were interested to find new antibacterial activities against Gram-positive bacteria by screening a *Planobispora rosea *ESAC library in *Streptomyces lividans *[11]. In particular, we isolated the clone SL-40 containing a *P. rosea *40 kb fragment with antibacterial activity against Gram-positive bacteria. Sequencing and BLAST analysis of the insert revealed 30 genes not directly relatable to genes from secondary metabolism biosynthesis. Finally, the sub-cloning revealed that a 3.5 kb fragment encoding a membrane protein (Imp) and an ABC transporter (Abc), whose role is unknown in *Planobispora*, was attributed to the antibacterial effects.

The presence of many cryptic or silent gene clusters in Streptomycetes [16-18] on one hand and the evidence that the two membrane proteins were both implicated in the antibacterial activity on the other hand suggested that the activity was due to an interaction between *Planobispora *cloned genes and *S. lividans *genes.

Similar effects have been described by Rondon *et al*. [19], who found that an antibacterial activity was determined by a membrane protein heterologously expressed in *E. coli *from a metagenomic library and their results suggested that this protein caused an effect on the host cell.

Unexpectedly, HPLC-ESI-mass spectrometry and HPLC-UV-DAD unambiguously showed that the antibacterial activity was due to thiostrepton, antibiotic used for recombinant clones selection. Furthermore, our findings revealed that a *de novo *thiostrepton synthesis had not occurred in *S. lividans *(Fig. 2) but strongly suggested that SL-3.5 antibacterial activity was caused by increased intracellular amounts of thiostrepton (Fig. 4) and its subsequent release in thiostrepton-free medium (Fig. 3). This effect could be explained by an increased uptake of thiostrepton from the medium, particularly triggered by expression of Imp and Abc (in addition to a entry by diffusion). *imp *and *abc *genes were found transcribed in a medium-dependent manner in *S. lividans*; in fact, their expression level was high enough to support the bioactivity only when the strain was grown in R_2_YED medium (Fig. 6b). Further analyses have to be carried out to elucidate the biological mechanism.

In the course of our experiments, the thiostrepton biosynthetic cluster of the producer strain *Streptomyces laurentii *was sequenced [5] and its absence in the SL-3.5 strain was in agreement with our results (Fig. 2).

Although best known as an inhibitor of protein synthesis, thiostrepton is also a potent activator of gene expression in *Streptomyces lividans*, i.e. it activates the expression of the so-called thiostrepton-induced proteins (TipAS and TipAL), whose role is still unknown. In the absence of added cofactors, thiostrepton was reported to form a highly stable complex with TipAL and TipAS in aqueous solution, which cannot be dissociated by denaturants such as SDS, urea, or disulfide reducing agents [20]. Our DIGE-analysis showed that large amounts of thiostrepton enter the cell, induce TipA expression and bind this protein, as demonstrated by the abundance of TipAS and TipAS-thio in the mycelium grown also in the absence of thiostrepton (Fig. 5). Thereby, the strain needs to grow for many generations in an antibiotic free medium in order to dilute the thiostrepton intracellular overstock in a appreciably way.

Moreover, the role of thiostrepton is not well understood; it is hypothesized that active thiopeptides are not only antibiotics but may serve as, or perhaps mimic, a signal molecule for physiological development or quorum-sensing in bacteria [1]. It was suggested that antibiotics are not only bacterial weapons for fighting competitors but also signaling molecules that may regulate the homeostasis of microbial communities [21]. For example, didehydroamino acids of thiostrepton are highly reactive toward low molecular weight thiols that maintain the cytoplasmic redox potential [22] and it seems likely that thiostrepton levels routinely used for selection of *tsr*-containing vectors could reduce the concentrations of these compounds and thereby disrupt overall cellular thiol-balance.

The expression of the two putative *P. rosea *membrane proteins, responsible for increasing intracellular amounts and storage of thiostrepton in *S. lividans*, causes a false antibiotic production, that creates errors in the interpretation of clone library screening results.

Our results showed that *S. coelicolor *containing *imp *and *abc *does not uptake thiostrepton, probably since the genes are transcribed at a low level (Fig. 6b). We can speculate that in *S. lividans *Imp and Abc serve as receptors or channels to up-take molecules such as thiostrepton. Notwithstanding, further analyses are necessary to investigate their role in *P. rosea *and in other heterologous hosts, as well.

## Conclusion

The heterologous gene expression has to be considered as an useful biotechnological tool not only for new antibiotic discovery but also to study gene function in amenable hosts. However, this work might result in the reconsideration of a careful screening of a genomic library directed to find new antibacterial substances especially when genes encoding membrane proteins are cloned and antibiotics with possible biological effects interfering with the physiological activity of the host, as thiostrepton, are used as clone selection markers.

## Methods

### Reagents

Thiostrepton was purchased from Fluka (Buchs, Switzerland).

### Strains, plasmids and growth conditions

The bacterial strains and plasmids used in this study are listed in Table 1. *Micrococcus luteus *and *Bacillus subtilis *were used for bioassays. Four clones (SL-40, -85, -48 and -120) were derived from an ESAC genomic library of *P. rosea *[11]. *S. lividans *strains were cultured in the following media: MG, JM, R_2_YED, ONA, TSB, Rare3 and R3 [14,23], according to Puglia *et al. *[24]. The SL clones were selected using thiostrepton (50 μg ml^-1^).

**Table 1:** Strains and plasmids.

Strain/Plasmid	Genotype/Description	Source/Reference
**Bacterial strains**		
*Planobispora rosea *ATCC53773		Biosearch Italia SpA, Gerenzano
*Streptomyces lividans *ZX7	*pro-2 str-6 rec-46 Δdnd HAU3*SLP2- SLP3-	John Innes Centre, Norwich, UK
*Streptomyces laurentii*		German Collection of Microorganisms and Cell Cultures, DSMZ GmbH, Braunschweig, Germany
*Streptomyces coelicolor M145*		John Innes Centre, Norwich, UK
*Escherichia coli *DH10B	F^- ^*mcrA Δ(mrr- hsdRMS-mcr BC) Δ80dlacZDM15 ΔlacX74 deoR recA1 endA1 araD139 Δ(ara, leu) 7697 galU GalK D*^- ^*rpsL nupG*	Life Technologies, Gibco BRL.
		
EC-40	*E. coli *containing ESAC40	Alduina *et al*., 2003
SC-3.5	*S. coelicolor *containing ESAC3.5	This study
SL-ESAC	*S. lividans *containing ESAC	Alduina *et al*., 2003
SL-40	*S. lividans *containing ESAC40	Alduina *et al*., 2003
SL-3.5	*S. lividans *containing ESAC3.5	This study
SL-Abc	*S. lividans *containing ESACAbc	This study
SL-Imp	*S. lividans *containing ESACImp	This study
SL-Abc/Imp	*S. lividans *containing ESACAbc and pNImp	This study
SL-Imp/Abc	*S. lividans *containing ESACImp and pNAbc	This study
		
**Plasmids**		
pUC	pUC18	
pBS	pBluescript	
ESAC	*Streptomyces*-*E. coli *Artificial Chromosome	Sosio *et al*., 2002
ESAC40	ESAC containing a *P.rosea *40 kb fragment	Alduina *et al*., 2003
ESAC3.5	ESAC containing a *P.rosea *3.5 kb fragment with *abc *and *imp *genes	This study (fig.1)
ESACAbc	ESAC3.5/*imp*::pBS	This study (fig.1)
ESACImp	ESAC3.5/*abc*::pUC	This study (fig.1)
pN702GEM3		Fernández-Abalos *et al*., 2003
pNAbc	pN702 containing *abc*	This study
pNImp	pN702 containing *imp*	This study

### Subcloning in *E. coli *and *Streptomyces *strains

Plasmid DNA, extracted from EC-40 by alkaline lysis method [25] and digested with *Bam*HI, was ligated to *Bam*HI-digested and dephosphorylated ESAC vector and the ligation mixture was used to transform *Escherichia coli *ElectroMAX DH10B cells, as described in Alduina *et al*., 2005 [12]. *Streptomyces *protoplast formation, transformation and regeneration were carried out according to the methods of Kieser *et al*. [14]. Southern hybridization was performed according to standard protocols [25]. DNA fragments used as probes were labelled with [α-^32^P]-CTP by the Rediprime II™ Random Prime Labelling System (Amersham Pharmacia Biotech, UK).

### Sequencing of the 40 kb DNA fragment from ESAC40

DNA sequencing was performed by MWG Biotech Laboratories (Ebersberg, Germany). Open reading frame (ORF) analysis was performed using Swiss prot protein knowledge base. Putative *orfs *were analyzed using BLAST against the NCBI non redundant protein database. The GenBank sequence accession number of the *Planobispora rosea *40 kb DNA fragment is EU908202.

### Disruption of *imp *and *abc*

The *imp *and *abc *disruption mutants were obtained by inserting pBluescript and pUC18 into the unique sites *Apa*I and *Sac*I site of ESAC3.5, respectively. The resulting ESAC*abc *and ESAC*imp *plasmids were used to transform *S. lividans *protoplasts, generating SL-Abc and SL-Imp clones, respectively. Genomic DNA extracted from these clones was digested with *Bam*HI and analyzed by Southern hybridization using the 3.5 Kb *Bam*HI fragment from ESAC3.5, containing both *orfs*, as a probe. As expected, two bands (6.5 and 6.2 Kb, respectively) were obtained for SL-Abc and SL-Imp (Fig. 1B, lane 5 and 6), while a 3.5 kb band was obtained for SL-3.5 (Fig. 1B, lane 1).

### Complementation of *imp *and *abc *mutations

A *Hind*III fragment containing *imp *and a *Bgl*II fragment containing *abc*, derived from ESAC3.5, were ligated into *Hind*III and *Bam*HI sites of pN702GEM3 [26] to obtain pN*Imp *and pNA*bc *plasmids, respectively. These plasmids were introduced by protoplast transformation in SL-Abc and SL-Imp to generate complementation strains, isolated under apramycin selection (100 μg/ml^-1^).

### Antibacterial assays and pre-purification of compounds

Antibacterial assays were carried out as follows: 5 ml of LB soft agar containing 100 μl of *M. luteus *suspension in water (OD_600 _of 1-1.2) were overlaid on LB agar plates. R_2_YED plugs of *S. lividans *strains, grown for four days at 30°C, were put on the *Micrococcus *overlay. Plates were then incubated overnight at 37°C and scored for activity by looking for an inhibition zone in the *M. luteus *lawn. The protocols reported in Hwang *et al*., 2003 [27] and Adamis *et al*., 1990 [28] were used for the detection of actinorhodin, undecylprodigiosin and calcium-dependent antibiotic. To identify the antibiotic substance, the methanolic extract of 20 agar plates for each analysed strain was dried *in vacuo *and dissolved in 1 l of distilled water. After XAD-chromatography (XAD-16 material, 300 ml column; 2 wash steps: 1 l H_2_O, 1 l 10% MeOH; elution with 500 ml acetone) and evaporation of the solvent, the extract was dissolved in EtOAc/Acetone/H_2_O (1/1/1) and both phases were analysed by HPLC-ESI-MS.

### HPLC-ESI-mass spectrometry

HPLC-ESI-MS and HPLC-UV-DAD experiments were carried out with a Q-Trap 2000 mass spectrometer from Applied Biosystems Deutschland GmbH (Darmstadt, Germany) (HPLC-ESI-MS), equipped with an 1100 series capillary HPLC system (HPLC-ESI-MS) or a 1100 series HPLC system (HPLC-UV-DAD) from Agilent Technologies Deutschland AG (Waldbronn, Germany) fitted with a Luna 3u C18(2) 100 Å, 50 × 1 mm column (HPLC-ESI-MS) or 50 × 4.5 mm column (HPLC-UV-DAD) purchased from Phenomenex, Aschaffenburg, Germany. Gradient 1: 25% B to 100% B in 10 min, gradient 2: 5% B to 100%B in 10 min (A: water, HPLC-MS grade, 0.1% formic acid, analytical grade; B: acetonitrile, HPLC-MS-grade, 0.1% formic acid, analytical grade, all solvents were purchased from Carl Roth GmbH & Co, Karlsruhe, Germany).

The upper phase of SL-3.5 water:acetone:ethyl acetate (1:1:1) extract contained a large peak in HPLC-ESI-MS and HPLC-UV-DAD that was absent in SL-ESAC. Furthermore, experiments confirmed its identity to thiostrepton purchased from Fluka (data not shown). Thiostrepton elutes at 7.0 min (HPLC-ESI-MS, gradient 1), 8.9 min (HPLC-ESI-MS, gradient 2) and 7.1 min (HPLC-UV-DAD, gradient 2), respectively and has a molecular mass of [M+H]^+ ^= 1664.5.

### Thin Layer Chromatography (TLC) and Feeding experiments

1 ml of pre-culture in JM medium of SL-ESAC, SL-3.5, SC-3.5, *Streptomyces coelicolor *and *S. laurentii *were streaked on R_2_YED medium and incubated at 30°C for four days before freezing at -20°C. The frozen mixture was added to 30 ml of methanol. An aliquot of 10-fold concentrated methanol extract was extracted with a mixture of water:acetone:ethyl acetate (1:1:1) and the upper phase was analyzed by Thin Layer Chromatography (TLC). TLC plates were developed in acetone and used for the bioassay. 0.050 μg/μl of thiostrepton in water:acetone:ethylacetate 1:1:1 were used as control.

In feeding experiments the isotopically labelled substrate (R,S)-[3-^14^C]cysteine (30 μCi ml^-1^) was added to R_2_YED medium and its presence in the extracts was detected by autoradiographic exposition of the TLC plate after acetone development.

### Analysis of thiostrepton in the mycelium of SL-3.5

SL-3.5, *S. laurentii *(a thiostrepton producer, positive control) and SL-ESAC (negative control) were each grown on R_2_YED agar plates containing thiostrepton (50μg/ml). After 4 days mycelium from all the three strains was collected and transferred to thiostrepton-free R_2_YED agar plates (passage I). Mycelium from this passage was subsequently transferred to new thiostrepton-free R2YED agar plates (passage II). This procedure was repeated 10 times. To facilitate mycelium isolation, cellophane was placed between the agar and the growing *Streptomyces *strains. After passages I, V and X of each strain, 0.16-0.17 g of mycelium were separated from agar and resuspended in 8 ml of distilled water. The suspension was sonicated three times on ice (15 sec in Vibra cell-output control 4) and the lysates were centrifuged for 10 min at 3000 r.p.m. Thiostrepton present in the pellet was extracted using 2 ml of DMSO and 5 μl of this extract were analysed by bioassay on *M. luteus *and visualization using an UV lamp (302 nm). The remaining solution was analyzed by HPLC-ESI-MS: the samples were freeze-dried and dissolved in 200μl DMSO, centrifuged and 4μl of the clear supernatant were injected into the HPLC-ESI mass spectrometer. The mass range during data acquisition was set to *m/z *1600 - 1680 and the peak at t_R _= 8.9 min and [M+H]^+ ^= 1664,5 Da corresponding to thiostrepton was integrated using the quantification tool in the MS-software *Analyst 1.4.2 *from Applied Biosystems. Prior to thiostrepton quantification a calibration curve was established using external samples of thiostrepton dissolved in DMSO in 10 different concentrations between 0.05 μg/ml and 20 μg/ml (y = 2332498.99 ×, R^2 ^= 0.97).

### Protein extraction, separation and analysis

Cells, grown as above, were recovered from cellophane and protein extraction was performed as described previously [11]. Protein concentration was quantified using the Qubit™ fluorometer (Invitrogen). After dialysis against distilled water at 4°C and acetone precipitation at -20°C, 300 mg of extracted protein was resuspended in 50 ml of lysis buffer (30 mM TRIS, 7 M urea, 2 M thiourea, 4% CHAPS) to carry out Two-Dimensional Differential Gel Electrophoresis (2D-DIGE, Amersham).

The pH of protein extracts was adjusted to 8.5 - 8.8 and 75 mg of proteins were labelled with CyDye DIGE fluors (Cy3 for SL-3.5 grown with or without thiostrepton and SL-Abc with thiostrepton; Cy5 for SL-ESAC with or without thiostrepton and SL-Abc without thiostrepton), according to the manufacturer's instruction. A pooled set of internal standards, comprising 25 mg aliquot from each sample, was minimally labelled with Cy2 DIGE fluors. After the labelling, rehydratation buffer was added to obtain 350 μl (final volume) before IEF separation. Proteins were first separated on 18 cm IPG strips (non-linear gradient pH 3-10) in the ETTAN IPGphor isoelectric focusing system. The IPG strips were rehydrated in IEF buffer containing 0.5% (w/v) ampholytes and 1% (w/v) bromophenol blue. For protein separation, a 30-V pre-step was performed for 10 h, followed by IEF carried out for 74,850 V-h with a maximum voltage of 8,000 V. All the steps were performed at 20°C using 50 μA per strip. After IEF, the IPG strips were saturated with an equilibration buffer (6 M urea, 30% (v/v) glycerol, 2% (w/v) SDS, 0.05 M TRIS-HCl pH 6.8 and 2% (w/v) DTE) for 12 min, in order to resolubilize proteins. The thiol groups of Cys were then blocked by substituting DTE with 2.5% (w/v) iodoacetamide in the equilibrating buffer. The focused proteins were then separated on 12% sodium dodecyl polyacrylamide gels (SDS-PAGE) at 16°C in a Hoefer Dalt vertical system, using a maximum setting of 40 μA and 110 V per gel. Two-dimensional gel analysis was performed using the DIGE algorithms of ImageMaster 2D platinum software (v. 6.01, GE Healthcare Biosciences) according to the manufacturer's instructions. Spot detection was performed automatically using the auto-detection device. Spot quantification was calculated as the volume of the spots (i.e. integration of optical density over the spot area). Spot volumes were normalized (%Vol) to the sum of the volume of all spots detected on each gel by the software. To better visualize the results, the relative values were reported as %Vol × 100.

### Total RNA isolation, RT-PCR analysis, and real-time RT-PCR

Collected mycelium of SL-3.5 and SC-3.5 grown on R_2_YED and ONA agar plates was broken by using 1 mg of lysozyme/ml in P-buffer and total RNA was extracted by using the RNeasy midi-kit (QIAGEN) and following the procedures reported in Alduina *et al*., 2007 [29]. As control of RNA quality, a reverse transcription-PCR (RT-PCR) with 0.1 μg of total RNA and primer pairs internal to *hrdB *(5'-cgtcgagggtcttcggctg-3' and 5'-cgcgagcccatctcgctgc-3') was carried out using a Superscript One-Step RT-PCR kit (Invitrogen) and the conditions indicated by the supplier. A negative control with *Taq *polymerase and without reverse transcriptase was included to assess the absence of chromosomal DNA.

Expression of *imp *and *abc *was analyzed quantitatively by real-time RT-PCR using the Applied Biosystems 7300 real-time PCR system (Applied Biosystems). Specific primers internal to *imp *(5'-tgccagcgcgtacaggtgtcg-3' and 5'-cgcgggtcttggtctggctgt-3') and *abc *(5'-cgtgcctggcccttcatcgac-3' and 5'-ggccctggcagatcatcgtgg-3') genes were designed using the web-based tool *GeneTool™ *[30]. For cDNA synthesis, 4 μg for each RNA were used as template in a 50 μl reaction volume employing High Capacity cDNA Archive Kit (Applied Biosystems, USA) according to the manufacturer's instructions. PCR cycling was performed at 25°C for 10 minutes, 37°C for 120 minutes and then 5 minutes at 85°C.

2.5 μl of each sample were used in the quantitative PCR reaction with SYBR^® ^Green PCR Master Mix (Applied Biosystems, USA) according to the manufacturer's instructions. Each 25 μl reaction contained 10 pmol of forward and reverse primers.

The PCR was performed under the following conditions: 2 min at 50°C and 10 min at 95°C, followed by 40 cycles of 15 s at 95°C and 1 min at 60°C. Eventually, a dissociation reaction was performed with the following conditions: a 1-min step with a temperature gradient increase of 1°C per step from 55 to 99°C. This last reaction allowed the melting curve of the PCR products and, consequently, to determine their specificity. A negative control (distilled water) was included in all real-time PCR assays, and each experiment was performed in triplicate. The *hrdB *(5'-cgtcgagggtcttcggctg-3' and 5'-cgcgagcccatctcgctgc-3') gene was used as an internal control to quantify the relative expression of target genes.

## Competing interests

The authors declare that they have no competing interests.

## Authors' contributions

AG carried out the construction of the mutants, feeding and real-time RT-PCR experiments and DIGE analysis and wrote the draft manuscript. RA carried out Southern analysis, participated in the design of the experiments and helped to draft the manuscript. EG carried out the chemical analysis and helped to draft the manuscript. VDC carried out the construction of *S. lividans *subclones. RDS participated in the design of experiments and revised the manuscript. AMP conceived the study and participated in its design and coordination and revised the manuscript.

All authors read and approved the final manuscript.

## References

[B1] Holmes DJ Caso JL Thompson CJ Autogenous transcriptional activation of a thiostrepton-induced gene in *Streptomyces lividans* EMBO J 1993 12 3183 3191 7688297 10.1002/j.1460-2075.1993.tb05987.x PMC413584

[B2] Chiu ML Folcher M Katoh T Puglia AM Vohradsky J Yun BS Seto H Thompson CJ Broad spectrum thiopeptide recognition specificity of the *Streptomyces lividans *TipAL Protein and its role in regulating gene expression JBC 1999 274 29 20578 20586 10.1074/jbc.274.29.20578 10400688

[B3] Murakami T Holt TG Thompson CJ Thiostrepton-induced gene expression in *Streptomyces lividans* J Bacteriol 1989 171 1459 1466 2537819 10.1128/jb.171.3.1459-1466.1989 PMC209767

[B4] Yun BS Hidaka T Kuzuyama T Seto H Thiopeptide non-producing *Streptomyces *species carry the tipA gene: a clue to its function J Antibiot 2001 54 4 375 378 11426662 10.7164/antibiotics.54.375

[B5] Kelly WL Pan L Li C Thiostrepton biosynthesis: Prototype for a new family of bacteriocins J Am Chem Soc 2009 131 12 4327 4334 10.1021/ja807890a 19265401

[B6] Liao R Duan L Lei C Pan H Ding Y Zhang Q Chen D Shen B Yu Y Liu W Thiopeptide biosynthesis featuring ribosomally synthesized precursor peptides and conserved posttranslational modifications Chem Biol 2009 16 2 141 147 10.1016/j.chembiol.2009.01.007 19246004 PMC2676563

[B7] Morris RP Leeds JA Naegeli HU Oberer L Memmert K Weber E LaMarche MJ Parker CN Burrer N Esterow S Hein AE Schmitt EK Krastel P Ribosomally synthesized thiopeptide antibiotics targeting elongation factor Tu J Am Chem Soc 2009 131 16 5946 5955 10.1021/ja900488a 19338336

[B8] Arndt HD Schoof S Lu JY Thiopeptide antibiotic biosynthesis Angew Chem Int Ed Engl 2009 48 37 6770 6773 10.1002/anie.200901808 19536800

[B9] Zhang H Wang Y Pfeifer BA Bacterial hosts for natural product production Mol Pharm 2008 5 3 472 10.1021/mp800045q 18232637

[B10] Alduina R Ferraro C Giardina A Di Caro V Sosio M Donadio S Puglia AM Del Vecchio V, Krcmery V Bacterial artificial chromosome libraries of antibiotic-producing actinomycetes Applications of Genomics and Proteomics for Analysis of Bacterial Biological Warfare Agents 2003 352 NATO Science Series. 111 121

[B11] Alduina R De Grazia S Dolce L Salerno P Sosio M Donadio S Puglia AM Artificial chromosome libraries of *Streptomyces coelicolor *A3(2) and *Planobispora rosea* FEMS Microbiol Lett 2003 218 181 186 12583916 10.1111/j.1574-6968.2003.tb11516.x

[B12] Alduina R Giardina A Gallo G Renzone G Ferraro C Contino A Scaloni A Donadio S Puglia AM Expression in *Streptomyces lividans *of *Nonomuraea *genes cloned in an artificial chromosome Appl Microbiol Biotechnol 2005 68 5 656 662 10.1007/s00253-005-1929-y 15821915

[B13] Selva E Beretta G Montanini M Saddler GS Gastaldo L Ferrari P Lorenzetti R Ripamonti F Goldstein BP Berti M Montanaro L Denaro M Antibiotic GE2270 a: a novel inhibitor of bacterial protein synthesis. Isolation and characterization J Antibiot 1991 44 7 693 701 1908853 10.7164/antibiotics.44.693

[B14] Kieser T Bibb MJ Buttner MJ Chater KF Hopwood DA Pratical *Streptomyces *genetics John Innes Centre, Norwich 2000

[B15] Chiu ML Viollier PH Katoh T Ramsden JJ Thompson CJ Ligand-induced changes in the *Streptomyces lividans *TipAL protein imply an alternative mechanism of transcriptional activation for MerR-like proteins Biochemistry 2001 40 43 12950 12958 10.1021/bi010328k 11669632

[B16] Bentley SD Chater KF Cerdeño-Tárraga AM Challis GL Thomson NR James KD Harris DE Quail MA Kieser H Harper D Bateman A Brown S Chandra G Chen CW Collins M Cronin A Fraser A Goble A Hidalgo J Hornsby T Howarth S Huang CH Kieser T Larke L Murphy L Oliver K O'Neil S Rabbinowitsch E Rajandream MA Rutherford K Rutter S Seeger K Saunders D Sharp S Squares R Squares S Taylor K Warren T Wietzorrek A Woodward J Barrell BG Parkhill J Hopwood DA Complete genome sequence of the model actinomycete *Streptomyces coelicolor *A3(2) Nature 2002 417 6885 141 147 10.1038/417141a 12000953

[B17] Ikeda H Ishikawa J Hanamoto A Shinose M Kikuchi H Shiba T Sakaki Y Hattori M Omura S Complete genome sequence and comparative analysis of the industrial microorganism *Streptomyces avermitilis* Nat Biotechnol 2003 21 5 526 531 10.1038/nbt820 12692562

[B18] Ohnishi Y Ishikawa J Hara H Suzuki H Ikenoya M Ikeda H Yamashita A Hattori M Horinouchi S Genome sequence of the streptomycin-producing microorganism *Streptomyces griseus *IFO 13350 J Bacteriol 2008 190 11 4050 4060 10.1128/JB.00204-08 18375553 PMC2395044

[B19] Rondon MR August PR Bettermann AD Brady SF Grossmann TH Liles MR Loiacono KA Lynch BA MacNeil IA other authors Cloning the soil metagenome: a strategy for accessing the genetic and functional diversity of uncultured microorganism App Environ Microbiol 2000 66 2541 2547 10.1128/AEM.66.6.2541-2547.2000 PMC110579 10831436

[B20] Chiu ML Folcher M Griffin P Holt T Thompson CJ Characterization of the covalent binding of Thiostrepton-induced protein from *Streptomyces lividans* Biochemistry 1996 35 2332 2341 10.1021/bi952073e 8652574

[B21] Linares JF Gustafsson I Baquero F Martinez JL Antibiotics as intermicrobial signaling agents instead of weapons PNAS 2006 113 51 19484 19489 10.1073/pnas.0608949103 PMC1682013 17148599

[B22] Newton GL Bewley CA Dwyer TJ Horn R Aharonowitz Y Cohen G Davies J Faulkner DJ Fahey RC The structure of U17 isolated from *Streptomyces clavuligerus *and its properties as antioxidant thiol Eur J Biochem 1995 230 821 825 10.1111/j.1432-1033.1995.0821h.x 7607257

[B23] Sosio M Giusino F Cappellano C Bossi E Puglia AM Donadio S Artificial chromosomes for antibiotic-producing actinomyces Nat Biotechnol 2000 18 343 345 10.1038/73810 10700154

[B24] Puglia AM Vohradsky J Thompson CJ Developmental control of the heat-shock stress regulon in *Streptomyces coelicolor* Mol Microbiol 1995 17 4 737 746 10.1111/j.1365-2958.1995.mmi_17040737.x 8801427

[B25] Sambrook J Fritsh EF Maniatis T Molecular cloning: a laboratory manual 1989 2 Cold Spring Harbor, NY: Cold Spring Harbor, Laboratory

[B26] Fernández-Abalos JM Reviejo V Díaz M Rodriguez S Leal F Santamaría RI Postranslational processing of the xylanase Xys1L form *Streptomyces halstedii *JM8 is carried out by serine proteases Microbiology-SGM 2003 149 1623 1632 10.1099/mic.0.26113-0 12855715

[B27] Hwang YS Kim ES Biró S Choi CY Cloning and analysis of a DNA fragment stimulating avermectin production in various *Streptomyces avermitilis *species App Environ Microbiol 2003 69 1263 1269 10.1128/AEM.69.2.1263-1269.2003 PMC143579 12571055

[B28] Adamis T Riggle P Champness W Mutation in a new *Streptomyces coelicolor *locus with globally block antibiotic biosynthesis but not sporulation J Bacteriol 1990 172 2962 2969 2345130 10.1128/jb.172.6.2962-2969.1990 PMC209095

[B29] Alduina R Lo L Piccolo D'Alia D Ferraro C Gunnarsson N Donadio S Puglia AM Phosphate-controlled regulator for the biosynthesis of the dalbavancin precursor A40926 J Bacteriol 2007 189 8120 9 10.1128/JB.01247-07 17873036 PMC2168674

[B30] Wishart DS Stothard P Van Domselaar GH PepTool and GeneTool: platform-independent tools for biological sequence analysis Methods Mol Biol 2000 132 93 113 10547833 10.1385/1-59259-192-2:93

